# Handgrip and contralateral knee flexion strength as key predictors of daily physical activity in severe knee osteoarthritis

**DOI:** 10.1002/jeo2.70540

**Published:** 2025-11-04

**Authors:** Daisuke Urashima, Tomofumi Kinoshita, Tatsuhiko Kutsuna, Kunihiko Watamori, Takashi Tsuda, Yusuke Horita, Kazunori Hino, Masaki Takao

**Affiliations:** ^1^ Department of Orthopaedic Surgery Ehime University Graduate School of Medicine Ehime Japan; ^2^ Department of Orthopaedic Surgery Kousei General Hospital Hiroshima Japan

**Keywords:** contralateral knee flexion strength, handgrip strength, knee muscle strength, knee osteoarthritis, physical activity monitor

## Abstract

**Purpose:**

In this study, factors associated with physical activity in patients with end‐stage knee osteoarthritis were investigated using a high‐precision wearable monitor. It was hypothesised that affected‐side knee muscle strength would significantly relate to objectively measured activity.

**Methods:**

A total of 58 patients with end‐stage knee osteoarthritis scheduled for total knee arthroplasty were recruited, between September 2024 and April 2025, and evaluated. Evaluations included handgrip strength, skeletal muscle index, and isokinetic knee extension and flexion strength. Daily physical activity was monitored for 1 month using a wearable device, which recorded the average time spent at different metabolic equivalent (MET) levels. Activity intensity was recorded in 0.1‐MET increments from 1.0 MET and categorised as 1.0–1.9, 2.0–2.9, 3.0–3.9, 4.0–4.9, 5.0–5.9, 6.0–6.9, 7.0–7.9 and ≥8.0 METs. Correlations were assessed by Spearman's rank coefficient; stepwise regression was performed adjusting for age, sex and body mass index.

**Results:**

The median duration of moderate‐to‐vigorous physical activity ( ≥3.0 METs) was 38.8 min/day. Using bivariate correlation analysis, handgrip strength and both affected‐ and contralateral‐side knee flexion and extension strength were significantly associated with the daily duration of activities at 4.0–4.9 METs (*ρ* = 0.397, *p* = 0.002; *ρ* = 0.276, *p* = 0.035; *ρ* = 0.364, *p* = 0.004; *ρ* = 0.302, *p* = 0.021; *ρ* = 0.368, *p* = 0.004, respectively). In contrast, skeletal muscle index was not significantly correlated with activity at any intensity level. Using stepwise multiple regression analysis, handgrip strength was a significant predictor of activities at 3.0–3.9 METs (*p* = 0.023), while contralateral knee flexion strength was a significant predictor at 4.0–4.9 METs (*p* = 0.003).

**Conclusion:**

In severe knee osteoarthritis, handgrip strength may screen patients at risk of reduced activity, while contralateral knee flexion strengthening in preoperative rehabilitation could help optimise management strategies.

**Level of Evidence:**

Level Ⅲ.

## Graphical abstract

Association between handgrip and knee muscle strength and physical activity in severe knee osteoarthritis

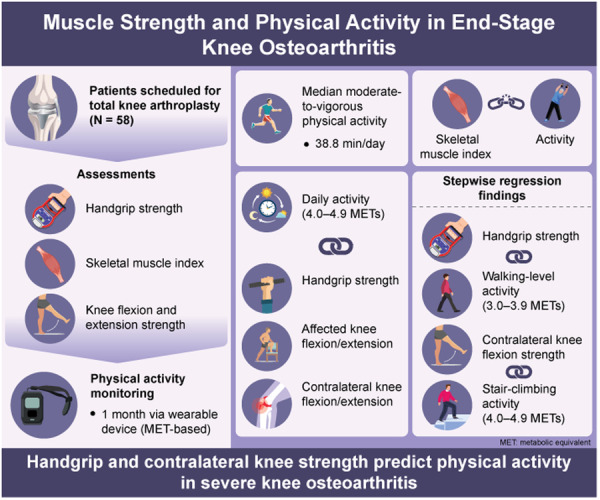

**Abbreviations**
ADL: activity of daily living
KOA: knee osteoarthritis
MET: metabolic equivalent
MVPA: moderate‐to‐vigorous physical activity
PROM: patient‐reported outcome measures
SMI: skeletal muscle index
TKA: total knee arthroplasty
WOMAC: Western Ontario and McMaster Universities Arthritis Index

## INTRODUCTION

Muscle weakness, particularly in the quadriceps and surrounding knee musculature, is a well‐recognised factor for the onset and progression of knee osteoarthritis (KOA) [6, 16, 18]. In addition to local deficits, systemic conditions, such as sarcopenic obesity, have also been reported as risk factors [27]. Muscle weakness is biomechanically detrimental and associated with increased pain and functional limitations; insufficient thigh muscle strength has even been suggested as a predictor for eventual surgical intervention such as total knee arthroplasty (TKA) [3]. Conversely, greater knee extensor strength has been linked to a reduced risk of joint space narrowing, underscoring its protective role [21]. Taken together, these findings highlight muscle strength as central to the prevention and management of KOA. However, in patients with severe KOA awaiting TKA, it remains unclear which specific muscle strength parameters or body composition measures are most relevant for sustaining daily physical activity.

Some studies have reported associations between muscle strength and physical activity in patients with mild‐to‐moderate KOA [2, 23]. Although most studies focused on knee extensor strength, some also emphasised the importance of knee flexors [23]. However, prior studies primarily relied on temporary outcome measures such as patient‐reported outcome measure or walking tests, which might not accurately reflect actual daily activity. Clarifying how muscle strength influences objectively measured daily activity would provide valuable insights into targeted interventions to maintain function and delay disease progression in patients with KOA.

To address this research gap, a high‐precision wearable activity monitor capable of capturing metabolic equivalent (MET) intensity in fine increments was employed to investigate the associations of handgrip strength, knee muscle strength, and skeletal muscle index (SMI) with intensity‐specific physical activity. It was hypothesised that knee muscle strength on the affected side and SMI could be associated with engagement in high‐intensity physical activities. Furthermore, identifying these associations might provide clinically relevant targets for individualised preoperative rehabilitation strategies in patients with severe KOA. Ultimately, these insights might contribute to the development of more effective, individualised preoperative rehabilitation strategies for managing patients with advanced KOA.

## METHODS

This prospective study was conducted in accordance with the principals of the Declaration of Helsinki and approved by the Institutional Review Board. Written informed consent was obtained from all patients. The study included patients scheduled for unilateral TKA at our institution and who did not have severe knee pain symptoms on the contralateral side. The participants were consecutively recruited between September 2024 and April 2025 from those who agreed to participate and provided informed consent. The exclusion criteria were: paralysis or neurological disorders, a prior surgical history of the affected knee joint, rheumatologic disease, or bilateral knee symptoms scheduled for simultaneous bilateral TKA or staged contralateral TKA. All patients had KOA with varus deformity. All knees had severe KOA, with Kellgren–Lawrence grade IV on the surgical side.

### Daily activities

**Figure 1:**
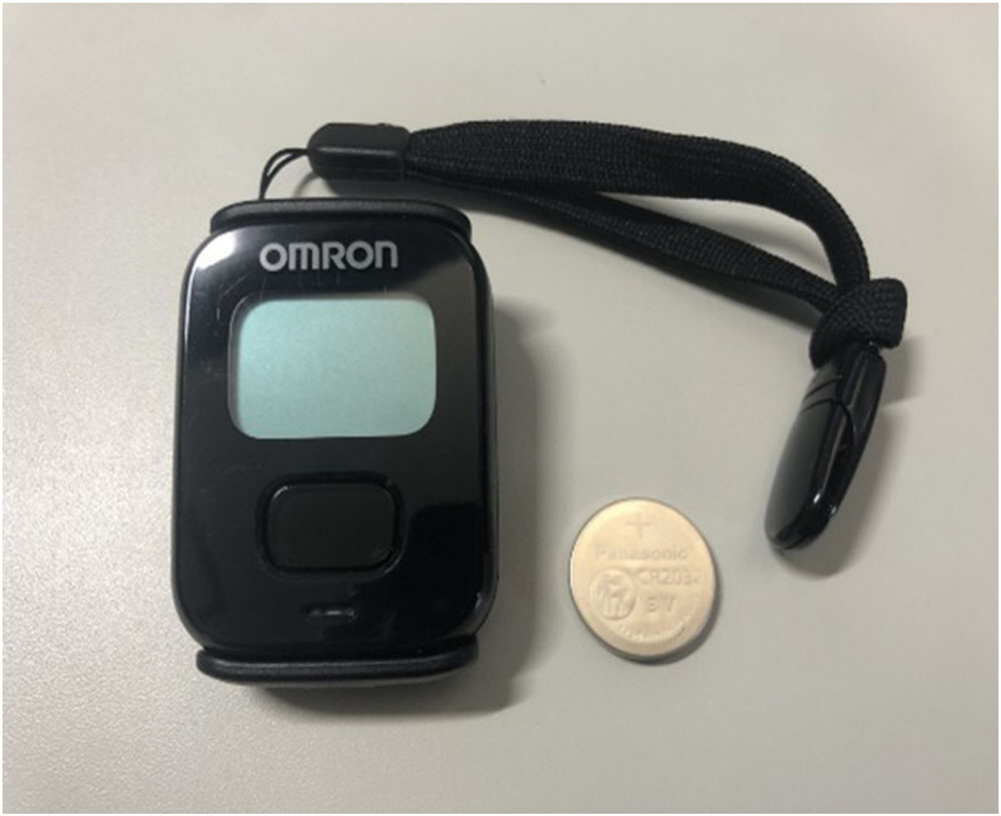
Wearable activity monitor with a lithium coin battery. It identifies walking and daily activities, recording physical activity data every 10 s to enable detailed data analysis.

In this study, daily physical activity was monitored using a wearable physical activity monitor (Active‐style Pro HJA‐750C, Omron Healthcare, Kyoto, Japan; Figure 1) for 30 consecutive days preoperatively as the primary outcome. This device records daily step counts and time spent in physical activities, classifying activity intensity in 0.1‐MET increments starting from 1.0 MET (Table 1) [13, 15, 17]. Regarding analysis, the detected activities were categorised into the following ranges: 1.0–1.9, 2.0–2.9, 3.0–3.9, 4.0–4.9, 5.0–5.9, 6.0–6.9, 7.0–7.9 and ≥8.0 METs. The participants were instructed to wear the device for 30 days; a valid day was defined as at least 10 h of wear time, according to previous studies [28]. We then calculated the average daily step count, average time spent in physical activities ≥1.0 METs, and average daily duration of moderate‐to‐vigorous physical activity (MVPA, ≥3.0 METs) on valid days. The accuracy and validity of this device have been confirmed in previous reports [13, 15, 17].

**Table 1:** Examples of exercise intensity for daily movement.

LPA (<3METs)	MVPA (≥3METs)
Sitting at a chair (1.0 METs)	Walking: 2.5mph (3.0 METs)
Ironing (2.0 METs)	Walking upstairs (4.7 METs)
Cooking (2.0 METs)	Walking: 4.0mph (5.0 METs)
Stretching and yoga (2.5 METs)	Jogging (7.0 METs)
Washing dishes (2.5 METs)	Running: 5 mph (8.3 METs)
Slow‐paced walking: 2.0 mph (2.8 METs)	Walking upstairs quickly (8.8 METs)

Abbreviations: LPA, light physical activity; METs, metabolic equivalents; MVPA, moderate‐to‐vigorous physical activity.

### Muscle strength and body composition

Handgrip strength was measured twice in each hand using a digital dynamometer; the maximum value was adopted for analysis. SMI was assessed once using a body composition analyser (InBody 770; InBody Co., Ltd., Seoul, Japan). Knee extension and flexion strength were assessed isokinetically using a dynamometer (Cybex NORM; Cybex International, USA). The participants performed five consecutive maximal knee extension/flexion movements; the peak torque value was adopted as the representative value. These measurements were assessed 1 day before TKA as secondary outcomes.

### Statistical analyses

The normality of each variable was tested using the Shapiro–Wilk test, which indicated that all physical activity measures were non‐normally distributed. Therefore, correlations between physical activity and muscle parameters (handgrip strength, SMI, and knee extension and flexion strength) were assessed by Spearman's rank correlation coefficient. To identify independent factors associated with physical activity, multiple linear regression analyses were performed with age, sex, and BMI fitted as fixed covariates, and muscle parameters as candidate predictors using a stepwise selection method. Non‐significant variables were automatically excluded from the model. Statistical significance was set at *p* < 0.05. All analyses were performed using SPSS Statistics version 28.0 (IBM Corp., Armonk, NY, USA). Since this was an exploratory observational study with a fixed number of available patients, an a priori power analysis was not performed.

## RESULTS

**Figure 2:**
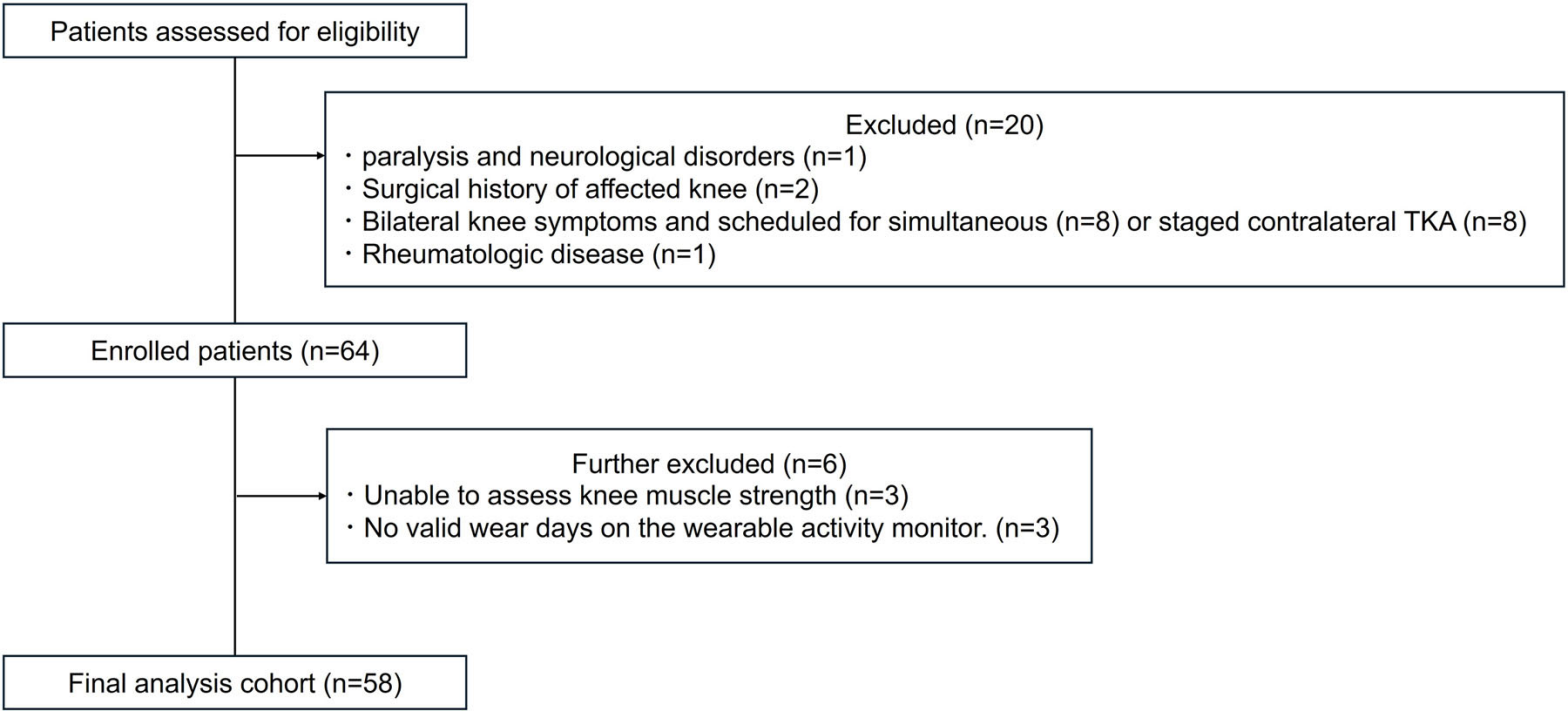
Flowchart of patient selection and inclusion in the final analysis cohort.

During the study period, 84 patients were screened. Among these, one patient with paralysis and neurological disorder, two with a prior surgical history of the affected knee, one with rheumatologic disease, and 16 with bilateral knee symptoms scheduled for simultaneous (*n* = 8) or staged contralateral TKA (*n* = 8) were excluded. In addition, six patients were excluded due to missing muscle strength assessment (*n* = 3) or insufficient valid wear days with the activity monitor (*n* = 3). Finally, data of 58 patients (10 men and 48 women) were included in the analysis (Figure 2). The baseline characteristics are presented in Table 2.

**Table 2:** Baseline characteristics of the study population.

	Mean ± SD
Age	64.4 ± 8.6 (45–87)
BMI	26.3 ± 4.2 (16.1–35.4)
HKA on affected side (°)	9.4 ± 6.0 (−13 to 24)
Maximum knee extension angle on the affected side (°)	−6.8 ± 6.4 (−25 to 0)
Maximum knee extension flexion on the affected side (°)	121.2 ± 10.2 (90–140)

*Note*: HKA, negative values indicate valgus and positive values indicate varus. For maximum knee extension angle, negative values indicate flexion contracture.

Abbreviations: BMI, body mass index; HKA, hip–knee–ankle angle; SD, standard deviation.

The median duration of MVPA ( ≥ 3.0 METs) was 38.8 min/day (Table 3). Table 4 presents the knee extension and flexion muscle strength using knee muscle strength measurements. Using the bivariate correlation analysis, the average daily duration of activities at 4.0–4.9 METs significantly correlated with knee flexion and extension muscle strength in both the affected and contralateral sides (*ρ* = 0.276, 0.364, 0.302 and 0.368; *p* = 0.035, 0.004, 0.021 and 0.004, respectively; Table 5) and handgrip strength (*ρ* = 0.397, *p* = 0.002; Table 6). In addition, the average daily duration of MVPA significantly correlated with knee flexion and extension muscle strength in the contralateral side and handgrip strength (*ρ* = 0.357, 0.266 and 0.365; *p* = 0.005, 0.043 and 0.004, respectively; Tables 5 and 6).

**Table 3:** Physical activity evaluated using a wearable physical monitor.

	Median (IQR)
Steps	1715.6 (653.9–3310.9)
MVPA (min)	38.8 (22.9–66.1)
1.0–1.9 METs (min)	517.9 (438.4–611.4)
2.0–2.9 METs (min)	157.8 (132.4–196.1)
3.0–3.9 METs (min)	35.2 (18.1–46.2)
4.0–4.9 METs (min)	5.2 (2.1–8.2)
5.0–5.9 METs (min)	1.3 (0.8–1.6)
6.0–6.9 METs (min)	0.4 (0.12–0.80)
7.0–7.9 METs (min)	0.02 (0–0.17)
≥ 8.0 METs (min)	0.03 (0–0.11)

Abbreviations: IQR, interquartile range; METs, metabolic equivalents; min, minutes; MVPA, moderate‐to‐vigorous physical activity.

**Table 4:** Knee extension and flexion muscle strength using isokinetic knee muscle strength measurements.

	Mean ± SD
Surgical side knee extension muscle strength	38.5 ± 19.0
Contralateral side knee extension muscle strength	45.1 ± 23.1
Surgical side knee flexion muscle strength	19.1 ± 14.4
Contralateral side knee flexion muscle strength	21.2 ± 14.6
Handgrip strength	22.3 ± 7.9
SMI	6.5 ± 1.4

Abbreviations: SD, standard deviation; SMI, skeletal muscle index.

**Table 5:** Relationship between preoperative knee muscle strength and physical activity.

Physical activity	Knee muscle strength							
	Surgical side knee extension		Contralateral side knee extension		Surgical side knee flexion		Contralateral side knee flexion	
	*ρ*	*p*‐Value	*ρ*	*p*‐Value	*ρ*	*p*‐Value	*ρ*	*p*‐Value
Age	−0.356	0.006**	−0.273	0.038*	−0.481	0.0001**	−0.324	0.013*
MVPA	0.246	0.062	0.357	0.005**	0.225	0.088	0.266	0.043*
1.0–1.9 METs	−0.061	0.649	−0.049	0.709	0.061	0.649	−0.013	0.917
2.0–2.9 METs	−0.003	0.981	0.109	0.413	−0.053	0.691	−0.126	0.345
3.0–3.9 METs	0.231	0.079	0.335	0.01*	0.202	0.127	0.228	0.083
4.0–4.9 METs	0.276	0.035*	0.364	0.004**	0.302	0.021*	0.368	0.004**
5.0–5.9 METs	0.196	0.138	0.269	0.04*	0.153	0.249	0.271	0.039*
6.0–6.9 METs	0.074	0.576	0.137	0.304	0.175	0.186	0.184	0.166
7.0–7.9 METs	0.003	0.980	−0.002	0.984	0.047	0.723	−0.075	0.574
≥ 8.0 METs	0.156	0.240	0.099	0.455	0.137	0.493	−0.104	0.603

*Note*: Surgical side knee extension, surgical side knee extension muscle strength; contralateral side knee extension, contralateral side knee extension muscle strength; surgical side knee flexion, surgical side knee muscle strength; contralateral side knee flexion, contralateral side knee flexion muscle strength.

Abbreviations: METs, metabolic equivalents; MVPA, moderate‐to‐vigorous physical activity.

* *p* < 0.05,

** *p* < 0.01.

**Table 6:** Relationship among handgrip strength, skeletal muscle index and physical activity.

	Handgrip strength		SMI	
	*ρ*	*p*‐Value	*ρ*	*p*‐Value
Age	−0.421	0.001**	−0.613	<0.0001**
MVPA	0.365	0.004**	0.139	0.295
1.0–1.9 METs	−0.045	0.737	0.017	0.894
2.0–2.9 METs	−0.071	0.592	−0.021	0.874
3.0–3.9 METs	0.347	0.007**	0.121	0.363
4.0–4.9 METs	0.397	0.002**	0.089	0.503
5.0–5.9 METs	0.245	0.0638	0.077	0.562
6.0–6.9 METs	0.103	0.441	0.071	0.596
7.0–7.9 METs	−0.068	0.609	0.022	0.150
≥ 8.0 METs	0.114	0.392	0.109	0.581

Abbreviations: METs, metabolic equivalents; MVPA, moderate‐to‐vigorous physical activity; SMI, skeletal muscle index.

** *p* < 0.01.

In the stepwise multiple regression analysis for the average daily duration of activities at 3.0–3.9 METs, handgrip strength was determined to be a significantly associated factor (*β* = 0.571, *p* = 0.023, 95% CI: 0.209–2.709, *R*² = 0.175; Table 7), whereas SMI and knee muscle strength variables were excluded from the model. Furthermore, regarding intensity levels of 4.0–4.9, contralateral knee flexion strength was identified as a significant associated factor, whereas SMI, handgrip strength, affected side knee extension and flexion strength and contralateral knee extension strength were excluded from the models (*β* = 0.487, *p* = 0.003, 95% CI: 0.080–0.365, *R*² = 0.329; Table 7). Using the stepwise multiple regression analyses, no explanatory variables remained in the models for the average daily duration of activities at 1.0–1.9, 2.0–2.9, 5.0–5.9, 6.0–6.9, 7.0–7.9 and ≥8.0 METs, indicating that none of the candidate factors were significantly associated with these outcomes.

**Table 7:** A stepwise multiple linear regression analysis between preoperative knee muscle strength and postoperative physical activity.

		*β*	95%CI	*p‐*Value
Average time at 3.0–3.9 METs intensity	Age	−0.211	−1.270 to 0.282	0.207
	Sex	0.349	−5.201 to 42.499	0.123
	BMI	−0.339	−2.995 to −0.281	0.019*
	Handgrip strength	0.571	0.209–2.709	0.023*
Average time at 4.0–4.9 METs intensity	Age	−0.294	−0.464 to 0.010	0.06
	Sex	0.107	−3.036 to 6.803	0.446
	BMI	−0.238	−0.783 to 0.026	0.066
	Contralateral side knee flexion muscle strength	0.487	0.080–0.365	0.003**

Abbreviations: *β*, unstandardised coefficient; BMI, body mass index; CI, confidence interval; METs, metabolic equivalents; MVPA, moderate‐to‐vigorous physical activity.

* *p* < 0.05,

** *p* < 0.01.

## DISCUSSION

This study evaluated factors related to physical activity in patients with end‐stage KOA using a high‐precision wearable physical activity monitor, and the primary finding is that, in patients with end‐stage KOA, handgrip strength predicted ambulatory‐level physical activity, whereas contralateral knee flexion strength was associated with engagement in moderate‐intensity physical activity. Although previous studies have primarily focused on knee extensor strength, the present findings suggest that, in patients with severe KOA requiring TKA, contralateral knee flexor strength may have a greater influence on ambulatory function in daily life. In contrast, SMI, which reflects whole‐body muscle mass, was not significantly correlated with physical activity levels in this population. This finding was of particular interest, as it contradicted the original hypothesis. To the best of our knowledge, this is the first study to concurrently evaluate SMI, handgrip strength, and both affected and contralateral knee flexor strength and investigate their associations with objectively measured physical activity using a wearable activity monitor.

In patients with KOA who experience severe pain and mobility limitations, muscle strength has been extensively reported as a critical determinant of physical function [2, 5, 23]. Chun et al. demonstrated that in patients with severe KOA, muscle strength was the only significant factor associated with performance deterioration [2]. In the present study including patients with advanced KOA scheduled for TKA, contralateral knee flexion strength was a significant determinant of daily physical activity. Greater contralateral strength may reduce the load on the affected knee and facilitate compensatory strategies that preserve functional capacity. Similarly, Bade et al. highlighted that postoperative contralateral limb function is a key determinant of rehabilitation outcomes after TKA [1].

Previous studies have identified specific muscle groups influencing function in patients with KOA. Preoperative quadriceps strength has consistently been shown to predict walking ability and activities of daily living (ADLs) [14], while Lopes et al. reported reduced hamstring strength in patients with KOA compared with healthy individuals, emphasising its role in rehabilitation [12]. Moreover, reductions in muscle output have been linked to clinically important declines in Western Ontario and McMaster Universities Arthritis Index (WOMAC) functional activity [19]. Furthermore, improvements in hamstring strength have been associated with better WOMAC functional scores, mobility, and stair‐climb performance [20]. The present study adds to this evidence by demonstrating that contralateral knee flexion strength is associated with moderate‐intensity physical activity in patients with severe KOA. In contrast to earlier research that primarily relied on PROMs or functional tests [8, 24, 25], the use of a wearable activity monitor enabled an objective evaluation of daily activity. These findings suggest that maintaining contralateral knee flexion strength is essential for preserving ADL in patients with severe KOA, consistent with reports indicating that preoperative activity levels facilitate early postoperative recovery [5].

In addition to periarticular knee muscles, broader indicators such as handgrip strength and sarcopenia have gained attention in recent years [27]. Sarcopenic obesity has been identified as a risk factor for the onset of KOA; [26, 27] delayed postoperative recovery has been observed in patients with sarcopenia [22], highlighting the relevance of systemic muscle assessment. Among these, handgrip strength has been linked to WOMAC functional activity [11] and to stair‐climbing ability after TKA [7]. In the present study, although SMI was not significantly associated with physical activity, handgrip strength showed significant correlation with ambulatory‐level activity (3.0–3.9 METs/day). This aligns with findings in previous reports indicating that functional outcomes are more strongly influenced by muscle quality than by muscle mass alone [4, 9, 10]. The relatively advanced age of our cohort might also have contributed to the lack of association between SMI and activity. Overall, handgrip strength might serve as a practical screening tool for identifying patients at risk of reduced ADL performance in routine clinical practice.

This study has some limitations. First, the sample size was relatively small, and the cohort consisted solely of patients with severe KOA, whose physical activity levels might vary widely. Since this was an exploratory observational study with a fixed number of available patients, an a priori power analysis was not performed, which might have limited the statistical power of the study. Second, the relationship between pain and physical activity was not assessed, despite pain being a potential confounding factor. Furthermore, the observation period was restricted to the preoperative phase; thus, future studies are needed to clarify how preoperative factors, including muscle strength, influence long‐term postoperative outcomes. Another limitation of this study is that preoperative rehabilitation programs were not standardised among the participants. Detailed information on the frequency, content, and participation in rehabilitation was not available for each individual. In addition, comorbidities and preoperative physical activity indices (e.g., UCLA activity score, ASA classification, frailty index), which might influence activity levels, were not systematically collected in this study. This lack of information might have introduced residual confounding and should be addressed in future research. The major limitation of this study is that the alignment and Kellgren–Lawrence grade of the contralateral knee were not evaluated in all cases. Although we selected patients scheduled for unilateral TKA who did not report pain symptoms in the contralateral limb, structural degeneration in the contralateral knee might still have influenced physical activity. Therefore, further validation in a larger cohort with detailed assessment of contralateral alignment and Kellgren–Lawrence grade is warranted to strengthen the robustness and generalisability of findings of this study. However, previous studies have reported that patients with KOA exhibit reduced hamstring strength during both isometric and concentric contractions compared with pain‐free controls [12]. Therefore, in the present study, which included patients without contralateral knee pain, the potential influence of contralateral deformity on the results is likely to have been minimal. Despite these limitations, the findings that handgrip strength predicted lower‐intensity activity and that contralateral knee flexion strength was linked to higher‐intensity levels are relevant because they might have clinical implications in optimising preoperative and perioperative rehabilitation strategies in patients undergoing TKA.

## CONCLUSION

In severe knee osteoarthritis, handgrip strength may screen patients at risk of reduced activity, while contralateral knee flexion strengthening in preoperative rehabilitation could help optimise management strategies.

## AUTHOR CONTRIBUTIONS

**Daisuke Urashima**: Writing–original draft; investigation. **Tomofumi Kinoshita**: Conceptualisation; methodology; investigation; formal analysis; writing–review and editing. **Kazunori Hino**: investigation; writing–review and editing. **Tatsuhiko Kutsuna**, **Kunihiko Watamori**, **Takashi Tsuda**, and **Yusuke Horita**: Investigation. **Masaki Takao**: Conceptualisation; writing–review and editing.

## CONFLICT OF INTEREST STATEMENT

The authors declare no conflict of interest.

## ETHICS STATEMENT

This study was approved by the Institutional Review Board of Ehime University (identification number: 2409007). All participants provided written informed consent.

## Data Availability

The data sets generated and/or analysed during the current study are available from the corresponding author on reasonable request.
